# The Large Jellyfish *Rhizostoma luteum* as Sustainable a Resource for Antioxidant Properties, Nutraceutical Value and Biomedical Applications

**DOI:** 10.3390/md16100396

**Published:** 2018-10-21

**Authors:** Laura Prieto, Angélica Enrique-Navarro, Rosalia Li Volsi, María J. Ortega

**Affiliations:** 1 Group Ecosystem Oceanography, Department of Ecology and Coastal Management, Instituto de Ciencias Marinas de Andalucía (CSIC), República Saharaui 2, 11519 Puerto Real, Cádiz, Spain; angelica.enrique@csic.es; 2 Department of Chemical, Biological, Pharmaceutical and Environmental Sciences, University of Messina, Viale F. Stagno D’Alcontres, 31, 98166 Messina, Italy; linda89lv@gmail.com; 3 Department of Organic Chemistry, University of Cádiz, Avda. República Saharaui s/n, 11510 Puerto Real, Cádiz, Spain

**Keywords:** cnidarians, gelatinous zooplankton, bioprospecting, novel foods

## Abstract

Jellyfish is a compartment in the marine food web that often achieves high increases of biomass and that it is starting to be explored for several human potential uses. In this paper, a recently rediscovered large jellyfish, *Rhizostoma luteum*, is studied for the first time to describe its organic compounds for the isolation and production of bioactive compounds in several fields of food, cosmetics, or biomedical industries. The biogeochemical composition (Carbon, Nitrogen and Sulfur content), protein and phenols content, together with their antioxidant activity, and the analysis of lipid content (identifying each of the fatty acids presented) was analyzed. The results presented here suggested this jellyfish has the highest antioxidant activity ever measured in a jellyfish, but also with high content in polyunsaturated fatty acids (PUFAs), including the essential fatty acid linoleic. The large natural biomass of *Rhizostoma luteum* in nature, the wide geographical spread, the fact that already its life cycle has been completed in captivity, establishes a promising positive association of this giant jellyfish species and the isolation of bioactive compounds for future use in marine biotechnology.

## 1. Introduction

One of the challenges for coming decades in nutraceutical research is to find new potential resources that will provide an easy and low-cost approach to food necessities for the coming centuries. In this sense, jellyfish is a compartment in the marine food web (the greatest biome on earth) that often achieves high increases of biomass [1]. However, only recently has this wide group of organisms been starting to be seen from a more positive point of view [2], contrasting with the deleterious impact that it has on several economic human activities such as fisheries [3], clogging of power plants [4] and tourism [5]. This dark side of jellyfish blooms has been the only vision that has prevailed in the general human perception for many decades [6]. One example of the new positive point of view of the rapid increment of biomass in jellyfish is the cannonball, *Stomolophus meleagris*, in the Gulf of California, where a small Mexican community obtains benefits from switching for one month from fishing fish to fishing jellyfish, processes them, and sells them to the Asiatic market [7]. Another example of the explosive increment of jellyfish is the case of *Cotylorhiza tuberculata* in the Mar Menor lagoon in the Mediterranean Sea, where during summer, fishermen are paid by regional authorities to catch jellyfish and bury them. Some summers they have caught more than five thousand tons of jellyfish [8,9].

The potential of jellyfish for human use is not only as a food resource [2,10,11,12,13], which is traditionally in Asian cuisine [14,15], but also recently as potential cosmeceutical and pharmacological applications [16]. Also, recently, jellyfish collagen has been studied for several biomedical applications [17], including antioxidant [18,19,20] and melanogenesis-inhibitory activity [18], skin photo-protection from Ultraviolet radiation [21,22], immunostimulatory effects [23,24], and antihypertensive effects [25].

The case we present here is unknown by the scientific community, and regards the potential use of the organic compounds and marine products from jellyfish. The jellyfish in the present work is *Rhizostoma luteum* Quoy and Gaimard, 1827 (Cnidaria: Scyphozoa: Rhizostomeae), which went scientifically unnoticed for more than 60 years until it was rediscovered and genetically characterized in 2012 [26]. Later, it was proved to be widely spread along the east coast of the Atlantic Ocean (from Portugal to South Africa) and in the Alboran Sea (Mediterranean Sea) and is much more frequent than previously thought, but was long misidentified for some of its congeners from the same geographical zones [27]. 

The purpose of the work is to describe the organic compounds for *Rhizostoma luteum* for the first time, with the hypothesis that it can be a candidate for the isolation and production of bioactive compounds in several fields of food, cosmetics, and biomedical industries. This hypothesis is based on three facts inherent to this jellyfish species: (1) it is from the family of the *Rhizostomaes*, which is the family of jellyfish preferable for consumption in Asiatic cuisine [28]; (2) The natural adult size of this jellyfish is very large, reaching more than 60 cm in umbrella size, weighing 13 kg and having 3 m tentacles [26,27,29], making one of the largest species of this family and therefore converting each individual into considerable amount of biomass; and (3) its life cycle has been recently described and closed entirely in captivity [29], which is a very difficult achievement since it is an open-ocean species. These three facts suggest that, in the case the potential use of *Rhizostoma luteum*, the exploitation of this species’ biomass can be obtained both from the natural environment and from controlled culture in captivity. However, before using it, it is mandatory to characterize the different organic compounds and other properties inherent to this jellyfish.

The main aim of this work is the identification of the primary metabolites (fatty acids) with antioxidant activity of the jellyfish *Rhizostoma luteum*. This goal was achieved by combining the original data on biochemical composition, protein, and phenol contents with their antioxidant activity, and the analysis of lipid content (identifying each of the presented fatty acids). The results presented here suggest this jellyfish to be a promising sustainable source for the production of several natural products. *Rhizostoma luteum* appears all year around on the coasts of the east Atlantic Ocean and Alboran Sea [29], providing a highly unexploited biomass. Now a positive association of this giant jellyfish species and an isolation of its bioactive compounds can be established.

## 2. Results and Discussion

### 2.1. Jellyfish Biomass Characterization

Biometric and average biomass data of individuals of *Rhizostoma luteum* are shown in Table 1. 

The specimens used are young medusae, with an age range from about 23–140 days (Table 1), with a proportionally increasing biomass with jellyfish age. The relation between fresh weight and diameter in *Rhizostoma luteum* has been described by [29], while the relationship between fresh weight (FW) and dry weight (DW) is documented for the first time for this species. The variability of the biometric measures, including the FW, the FW:diameter ratio and DW percentage values, is representative of the different growth stages of the jellyfish [30]. After lyophilization, the DW of *R. luteum* ranges from 3.9–31% of the FW, displaying a high variability in tissue consistency. The highest values correspond with the older jellyfish employed in this study.

The first parameter that should be assessed for evaluation in its potential use as biomass is the total energy or gross energy value, which is directly related to DW. The DW of *R. luteum* is similar to the measured range of the FW of *Cotylorhiza tuberculata* (3.9–32.4%), which is higher than *Aurelia* sp. 1 (2.2–3%) and *Rhizostoma pulmo* (4.1–6.8%) [16]. These values provide important information for the potential exploitation of *R. luteum* jellyfish biomass. It has recently been documented using different techniques that jellyfish may practically represent the total diet of several organisms, such as leatherback sea turtles and several fish [31,32,33,34].

### 2.2. Jellyfish Carbon, Nitrogen and Sulfur Content

The carbon, nitrogen and sulfur content of the jellyfish *Rhizostoma luteum* were 5.9 ± 0.3%, 1.6 ± 0.1% and 1.9 ± 0.2% of DW, respectively (mean ± standard deviation, *n* = 6 (Batch numbers: 3, 5–9)). The C:N molar ratio was 3.7 ± 0.1%. Similar values of carbon and nitrogen content have been reported in various of jellyfish such as *Aurelia aurita*, *Chrysaora fuscenses* and *Cyanea capillata* [35] and to the symbiotic jellyfish *Cassiopea xamachana* [36,37]. The C:N molar ratio of *R. luteum* is in the same range as overall medusae and ctenophores [38], and as the symbiotic adults of *Cotylorhiza tuberculata* [39] and *Cassiopea xamachana* [36,37]. 

### 2.3. Jellyfish Protein Content

The total freeze-dried tissue of 8 different batches were subjected to phosphate-buffered saline (PBS) solvent extraction treatment. The whole tissues of *Rhizostoma luteum* contained proteins soluble in polar solvents, specifically in aqueous solution with a mean value of 13.7 ± 3.4 mg of proteins of per g of dry weight (mean ± standard deviation, *n* = 8 (Batch numbers: 1, 2, 4–7, 9, 10)). The values ranged between 8.4–18.7 mg of proteins/g of DW. Compared to other species, such as *Aurelia* sp. 1, *Cotylorhiza tuberculata* and *Rhizostoma pulmo*, with 22, 35 and 37 mg of proteins/g of DW, respectively [16], *R. luteum* contained less soluble protein but in the same order of magnitude. The content of protein in jellyfish is consistently the most abundant organic fraction [38] and has been studied in other rhizostomae jellyfish such as *Rhopilema asamushi* [40] and from *Cyanea nozakii* [41] for collagen isolation and characterization, which has been suggested for multipurpose uses in cosmetics and nutraceutical sectors [42].

### 2.4. Phenolic Compound Content in Jellyfish Aqueous Soluble Extract

The total phenolic content of the jellyfish *Rhizostoma luteum* was 1964.9 ± 386.4 µg GAE (Gallic Acid Equivalent) per gram of DW in the PBS extract (mean ± standard deviation, *n* = 8 (Batch numbers: 1, 2, 4–7, 9, 10)). The values ranged between 1289.6–2597.1 µg GAE/g DW. The phenolic content in jellyfish is not well documented. Compared to *Aurelia* sp. 1 with 116 µg GAE/g DW in the PBS extract [16], *R. luteum* is in a position between *C. tuberculata* and *R. pulmo* reaching 1818 and 2079 µg GAE/g DW, respectively [16]. Also, phenolic compounds were detected in the podocyst and adults of *Chrysaora quinquecirrha* [43,44] and in *Cyanea capillata* and *C. tuberculata* [30,44]. 

The biostability and biochemical properties of collagen-based tissues may be enhanced by the polyphenols through modulation of mechanisms of collagen fiber cross-linking at different levels (molecular, inter-molecular and inter-microfibrillar) [45,46]. The high phenolic content of *R. luteum* (this study), *C. tuberculata* and *R. pulmo* [16], all of them large rhizostomae jellyfish, may be the reason for the robust and hardened mesoglea, compared to the flexible and soft *Aurelia* spp. 

### 2.5. Antioxidant Activity in Jellyfish Aqueous Soluble Extract

The total antioxidant activity of the jellyfish *Rhizostoma luteum* was 32,598 ± 9015 nmol of TE (Trolox Equivalent) per gram of DW in the PBS extract (means ± standard deviation, *n* = 8 (Batch numbers: 1, 2, 4–7, 9, 10)). The values ranged between 15,468–43,501 nmol of TE/g DW. These values of *R. luteum* are the highest values measured in a jellyfish so far, but in the same magnitude as other rhizostomae jellies such as *R. pulmo* and *C. tuberculata*, with antioxidant activity of 22,520 and 25,621 nmol of TE/g DW, respectively [16]. Meanwhile, *Aurelia* sp. 1 antioxidant activity was much lower, with measured values of 7651 nmol of TE/g DW [16]. 

The high values of *R. pulmo* and *C. tuberculata* compared to *Aurelia* sp. 1 were presumably attributed to the protein and phenols contents, although other unidentified compounds could be included [16]. Similarly, high values of antioxidant activity present in *R. luteum* in the present study could also be related to the protein and phenol content. The antioxidant activity referring to protein content, both in PBS extract, for *R. luteum* is 2379 nmol of TE/mg of proteins, showing evidence that this high antioxidant activity could be ascribed to the inherent protein properties of this jellyfish. Values of this ratio are double those reported by *R. pulmo* and *C. tuberculata* [16].

### 2.6. Lipid Content

Total lipid content of the jellyfish *Rhizostoma luteum* was around 0.94 g per 100 g of DW (standard deviation = 0.03, *n* = 6 (Batch numbers: 3, 5–8, 10)). This value was one order of magnitude lower than both *Aurelia* sp. 1 and *R. pulmo* (4 g/100 g DW) and two orders of magnitude lower than *C. tuberculata* (12.3 g/100 g DW; [16]). The low values of *R. luteum* are probably due to the age of the specimens examined. In the case of symbiotic jellyfish such as *C. tuberculata*, the high lipid content could be related to the photosynthetic membranes of the zooxanthellae [30,47].

The fatty acid (FA) quantitative composition (as percentage values) of *R. luteum* showed that polyunsaturated fatty acids (PUFA) accounted for half of the total FA (49%), followed by saturated fatty acids (SFA), representing one-third of the total FA (about 30%), and finally monounsaturated fatty acids (MUFA), representing around 21% of the total FA (Table 2). In the case of *Aurelia* sp.1, *C. tuberculata* and *R. pulmo*, the proportions were different to *R. luteum*, with SFA being the most abundant FA (two-thirds of the total FA), followed by PUFA (two-thirds of the total FA) and a small amount of MUFA (4–15%) [16]. 

Polyunsaturated fatty acids of *R. luteum* consisted mostly of ω-*6* arachidonic (C_20:4_), ω-*3* linoleic (C_18:3_) and the essential ω-*6* linoleic (C_18:2_) acids. Saturated fatty acids consisted mostly of stearic (C_18:0_) and palmitic (C_16:0_) acids. Among MUFA, oleic acid (C_18:1_) was the prevalent FA (Table 2). This composition of fatty acids shows remarkable differences to the other three species studied previously (see Table 4 in [16]). The proportion of PUFA of both ω-*6* arachidonic (C_20:4_) and ω-*3* linoleic (C_18:3_) in *R. luteum* are the highest documented for jellyfish and the essential ω-*6* linoleic (C_18:2_) acid is only topped by the symbiotic jellyfish *C. tuberculata* [16], giving a peculiar composition of PUFA in *R. luteum*. In the case of MUFA oleic acid, (C_18:1_) is in the same order of magnitude with *C. tuberculata* and higher than *Aurelia* sp.1 and *R. pulmo* [16]).

Both ω-*6* and ω-*3* PUFAs were abundant in the jellyfish *Rhizostoma luteum*, with the ratio of ω-*6* to ω-*3* of 2.14. The Σ ω-*6* was 33.4% while the Σ ω-*3* was 15.6%. The total ω-*3* PUFAs in *R. luteum* is similar to *Aurelia* sp. 1, *C. tuberculata* and *R. pulmo* [16], but the total ω-*6* PUFAs in *R. luteum* is double the other three documented jellyfish. Therefore, the ratio ω-*6* to ω-*3* of the other jellyfish is lower (between 0.4 to 0.8) [16] than in *R. luteum*. The role of ω-*3* PUFAs in a diverse biological process is well known, including development, growth, tissue, and cell homeostasis [48]. Regarding the numerous human health benefits of ω-*3* PUFAs, these include antiarthritis, anti-inflammatory, antioxidant, antihypertensive, anticancer, hypo-trigleceridemic, antiaging and antidepressive effects [49]. A high ratio of ω-*6* to ω-*3* in the Western diet in humans appears to be related to pathogenesis of chronic disease due to its proinflammatory effects [50]. The fatty acid composition of *R. luteum* discovered in this study should be considered for new applications due to its nutraceutical value. 

## 3. Materials and Methods 

### 3.1. Materials and Chemicals

Methanol and ethanol were purchased from Merck (Darmstadt, Germany); potassium persulfate (dipotassium peroxodisulfate), 6-hydroxy-2,5,7,8-tetramethylchroman-2-carboxylic acid (Trolox), 2,2’-azinobis(3-ethylbenzothiazoline-6-sulfonic acid) diammonium salt (ABTS), gallic acid, PBS, Folin-Ciocalteu’s phenol reagent, Bradford reagent, fatty acid methyl esters (FAME) Supelco 37 Component Mix were all purchased from Sigma-Aldrich Química (Madrid, Spain). All other reagents were of analytical grade. 

### 3.2. Sample Collection and Preparation

Specimens of *Rhizostoma luteum* jellyfish were reared in the laboratory (ICMAN-CSIC) from polyps. The origin of this living collection is from planulae gathered directly from the gonad of a free-swimming female medusa offshore of the coast of the Alboran Sea (Spain) in October 2015 (details in Kienberger et al. [29]). For the present study, a total of 303 specimens were used. After the biometric measurements (weight and diameter) were taken, and due to their small size, specimens were grouped to reach enough biomass to be analyzed and lyophilized. Samples were freeze-dried for 4 days at −55 °C using a chamber pressure of 0.110 mbar in a freeze dryer (LyoAlfa 15, Telstar Life Science Solutions, Madrid, Spain). Lyophilized samples were weighed to annotate the dry weight and stored at −20 °C until use. 

### 3.3. Elemental Analysis

Total carbon, nitrogen, and sulfur (C, N, S) content (in wt.%, approximately 100 mg per sample) were measured using an Elementary Chemical Analyzer LECO CHNS-932.

### 3.4. Polar Solvent Extraction

Lyophilized samples of total jellyfish were subjected to extraction in aqueous solvent PBS. Samples were stirred with 16 volumes of PBS (2 h at 4 °C). Samples were then centrifuged at 9000× *g* for 30 min at 4 °C and the supernatants were essayed for protein and phenol contents and for antioxidant activity. 

### 3.5. Protein Content 

Total protein content was estimated by modified Bradford assay [51] using bovine serum albumin (BSA) as a standard. 

### 3.6. Phenol Content 

The total phenolic content was determined by using a modified Folin-Ciocalteau colorimetric method. Aliquots of extracts (100 μL) were mixed with 500 μL of Folin-Ciocalteu’s phenol reagent and 500 μL of 7.5% sodium carbonate (Na_2_CO_3_). After 2 h of incubation at room temperature in the dark, the absorbance was spectrophotometrically measured at 760 nm. Gallic acid, ranging from 2.5 to 100 μg/mL, was used as a standard. The results were expressed as gallic acid equivalents (GAE) per gram of dry extract.

### 3.7. Antioxidant Activity 

The total antioxidant activity was determined spectrophotometrically by the ABTS free radical decolorization assay developed by Re et al. [52], with some modifications. In brief, a solution of the radical cation ABTS^+^ was prepared by mixing a solution of ABST (2,2’-azinobis(3-ethylbenzothiazoline-6-sulfonic acid) diammonium salt (7 mM) and a solution of potassium persulfate (2.45 mM) in H_2_O. The mixture was kept in the dark overnight before use. Then, the ABTS^+^ solution was diluted with EtOH to an absorbance of 0.70 ± 0.02 at 734 nm. Trolox was used as standard. The samples were prepared by adding 100 μL of extracts to 2 mL of ABTS^+^ solution and the control by adding 100 μL of PBS solution to 2 mL of ABTS^+^ solution. The measure at the absorbance at 734 nm was registered after 6 min of reaction. The percentage of inhibition of the absorbance was calculated by the following equation:

%inhibition = ((A_0_ − A_1_)/A_0_) × 100 (1)

where A_0_ is the absorbance of the control and A_1_ is the absorbance of the tested samples. Results were expressed as nmol of Trolox equivalents (TE) per gram of sample.

### 3.8. Total Lipid Extraction 

Total lipids were extracted using the modified Bligh and Dyer method [53]. Lyophilized powder (100 mg) was mixed with a total of 10 mL solvent added in the sequence of chloroform, methanol, water, to achieve a final chloroform/methanol/water ratio of 1:2:0.8 (by volume). Samples were shaken for 15 s after addition of each solvent, and incubated overnight at 4 °C. After centrifugation at 6500× *g* for 10 min, the supernatant was transferred into a separating funnel, and phase separation of the biomass-solvent mixtures was achieved by adding chloroform and water to obtain a final chloroform/methanol/water ratio of 2:2:1.8 (by volume). After settling, the bottom phase was collected and evaporated under vacuum. 

#### Fatty Acid Profiles Determination

Fatty acid methyl esters (FAMEs) were obtained using boron trifluoride (BF_3_) according to [54] with some modifications. Total lipid extract was saponified at 90 °C for 20 min with 0.5 M KOH in methanol (3 mL). Forty-nine micrograms of the internal standard (methyl tricosanoate) were added before saponification. The fatty acids were methylated by adding 14% BF_3_ in MeOH (2 mL) and heating at 90 °C for 10 min. After cooling, the mixture was extracted with hexane (1 mL × 2). After separation, the hexane layer was collected, taken to dryness under vacuum, dissolved in 1.0 mL of CH_2_Cl_2_ and analyzed by gas chromatography-mass spectrometry (GC-MS). 

### 3.9. GC-MS Analysis 

The analyses were performed on a GC-MS system consisting of high-resolution SYNAPT G2 (Waters, Milford, MA, USA) instrument equipped with a quadrupole-time-of-flight (QTOF) analyzer using atmospheric pressure ionization (API) in positive ionization mode. Compounds were separated on a DB-5 capillary column Agilent J&W DB-5ms column (250 µm × 30 m, 0.25 µm film) (Agilent, Santa Clara, CA, USA). The GC parameters were as follows: the column temperature was maintained at 90 °C for 1 min, then raised to 200 °C (20 °C/min) and to 300 °C (5 °C/min), and held at 300 °C for 2 min. Fatty acids were identified by comparison of retention time, molecular formula obtained by their high-resolution molecular ion [M + H]^+^, and mass spectral data with FAME standards (Supelco-37) and a NIST library.

## 4. Conclusions

The potential use of the jellyfish *Rhizostoma luteum* as biomass, evaluating their nutraceutical value and antioxidant properties, has never been evaluated until the present study. The results presented here suggest this jellyfish to be an excellent candidate for the potentially sustainable production of nutraceutical, cosmeceutical and biomedical natural products due to the highest antioxidant activity ever measured in a jellyfish, but also for their high content in PUFAs, including the essential fatty acid linoleic. Because the present work has been performed with young medusae, further work should be carried out with adult specimens. The large natural biomass of *Rhizostoma luteum* in nature, the wide geographical spread, and the fact that already its life cycle has been completed in captivity establishes a promising positive potential of this giant jellyfish species and the isolation of bioactive compounds for future use in marine biotechnology.

## Figures and Tables

**Table 1:** Biometric measures, fresh and dry weights of the different batches of *Rhizostoma luteum*.

Batch (*n* = Number of Jellyfish)	Days After Ephyra Release Range * Mean (days)	Umbrella Diameter Range * Mean (cm)	Fresh Weight Total (g)	Fresh Weight Per Individual Mean (g)	Ratio FW/Diameter	Dry Weight Total (g)	DW (% of FW)
1 (5)	109 ± 10	0.92 ± 0.03	2.9383	0.587 ± 0.106	0.6	0.912	31.0
2 (5)	92 ± 3	0.87 ± 0.01	3.0509	0.610 ± 0.121	0.7	0.808	26.5
3 (10)	89 ± 17	0.85 ± 0.06	9.6241	0.962 ± 0.142	1.1	0.350	3.6
4 (10)	85 ± 21	0.84 ± 0.07	5.6126	0.561 ± 0.144	0.7	0.2034	3.6
5 (10)	66 ± 10	0.77 ± 0.04	5.6181	0.560 ± 0.106	0.7	0.2747	4.9
6 (10)	72 ± 3	0.79 ± 0.01	4.5403	0.454 ± 0.086	0.6	0.1519	3.3
7 (41)	51 ± 22	0.69 ± 0.10	12.3213	0.300 ± 0.057	0.4	0.4609	3.7
8 (40)	42 ± 6	0.66 ± 0.04	5.9973	0.150 ± 0.028	0.2	0.1917	3.2
9 (61)	35 ± 6	0.62 ± 0.03	7.0676	0.116 ± 0.022	0.2	0.2384	3.4
10 (59)	51 ± 6	0.71 ± 0.03	9.9585	0.169 ± 0.032	0.2	0.388	3.9
11 (52)	53 ± 5	0.71 ± 0.03	8.7219	0.168 ± 0.032	0.2	0.332	3.8

FW, fresh weight; DW, dry weight; * data are expressed as range and/or means standard deviation (5 < *n* < 61).

**Table 2:** Fatty acid composition of *Rhizostoma luteum* expressed as percentage of the total fatty acid ± standard deviation (SD).

Type of Fatty Acids (FA)	Name of FA	RT (min)	%	Total
*Saturated FA (SFA)*	Decanoic acid C_10:0_	5.67	0.1 ± 0.0	30.2
	Lauric acid C_12:0_	8.65	0.3 ± 0.0	
	Tridecanoic acid C_13:0_	10.20	0.1 ± 0.0	
	Myristic acid C_14:0_	11.73	2.0 ± 0.1	
	Pentadecanoic acid C_15:0_	13.21	0.4 ± 0.1	
	Palmitic acid C_16:0_	14.65	11.0 ± 0.6	
	Margaric acid C_17:0_	16.03	0.7 ± 0.1	
	Stearic acid C_18:0_	17.40	15.0 ± 1.5	
	Arachidic acid C_20:0_	19.90	0.1 ± 0.0	
	Heneicosanoic acid C_21:0_	21.13	0.1 ± 0.0	
	Docosanoic acid C_22:0_	22.30	0.2 ± 0.0	
	Lignoceric acid C_24:0_	24.90	0.2 ± 0.0	
*Monounsaturated FA (MUFA)*	Pentadec-10-enoic acid C_15:1_ (ω5)	12.97	0.1 ± 0.1	20.8
	Palmitoleic acid C_16:1_	14.34	3.1 ± 0.2	
	Heptadec-10-enoic acid C_17:1_ (ω7)	15.70	0.4 ± 0.0	
	Oleic acid C_18:1_ (ω9)	17.02	11.3 ± 0.8	
	Elaidic acid C_18:1_ (ω9)	17.09	3.9 ± 0.1	
	Eicos-11-enoic acid C_20:1_ (ω9)	19.58	1.0 ± 0.0	
	Erucic or cis-docos-13-enoic acid C_22:1_ (ω9)	22.07	0.1 ± 0.0	
	Nervonic acid C_24:1_ (ω9)	24.54	0.8 ± 0.0	
*Polyunsaturated FA (PUFA)*	Gamma-linolenic acid C_18:3_ (ω6)	16.69	0.2 ± 0.0	49.0
	Linoleic acid C_18:2_ (ω6) *	16.92	4.6 ± 0.5	
	Linolelaidic acid C_18:2_ (ω6, ω9)	17.02	1.0 ± 0.1	
	Linolenic acid C_18:3_ (ω3)	17.02	9.8 ± 0.3	
	Arachidonic acid C_20:4_ (ω6)	19.02	23.7 ± 1.3	
	Eicosapentaenoic acid C_20:5_ (ω3)	19.08	3.6 ± 0.2	
	Dihomo-gamma-linolenic acid C_20:3_ (ω6)	19.25	3.2 ± 0.4	
	Eicosadien-11,14-oic acid C_20:2_ (ω6)	19.50	0.7 ± 0.0	
	Eicosa-11,14,17-trienoic acid C_20:3_ (ω3)	19.58	1.9 ± 0.1	
	Docosahexaenoic acid C_22:6_ (ω3)	21.36	0.3 ± 0.1	

RT: retention time; SFA: saturated fatty acids; MUFA: monounsaturated fatty acids; PUFA: polyunsaturated fatty acids; *: essential fatty acids. *n* = 6.
